# Elucidating the Molecular Mechanisms and Comprehensive Effects of Sludge‐Derived Plant Biostimulants on Crop Growth: Insights from Metabolomic Analysis

**DOI:** 10.1002/advs.202404210

**Published:** 2024-11-14

**Authors:** Yu Hua, Shuxian Chen, Tong Tong, Xiaoou Li, Rongting Ji, Qiujin Xu, Yue Zhang, Xiaohu Dai

**Affiliations:** ^1^ State Key Laboratory of Pollution Control and Resources Reuse College of Environmental Science and Engineering Tongji University Shanghai 200092 China; ^2^ State Key Laboratory of Environmental Criteria and Risk Assessment Chinese Research Academy of Environmental Sciences Beijing 100012 China; ^3^ Nantong Yuezichun Biological Agriculture Technology Co., Ltd Nantong 226000 China; ^4^ Nanjing Institute of Environmental Science Ministry of Ecology and Environment of the People's Republic of China Nanjing 210042 China; ^5^ China Civil Engineering Society Water Industry Association Beijing 100082 China

**Keywords:** alkaline thermal hydrolysis, land utilization, plant hormones, sewage sludge, widely targeted metabolites

## Abstract

The utilization of urban waste for land management plays a crucial role in reshaping material flows between human activities and the environment. Sewage sludge alkaline thermal hydrolysis (ATH) produces sludge‐derived plant biostimulants (SPB), which have garnered attention due to the presence of indole‐3‐acetic acid. However, there remains a gap in understanding SPB's molecular‐level effects and its comprehensive impact on crops throughout their growth cycle. In this study, non‐targeted and targeted metabolomic approaches are employed to analyze 51 plant hormones and 1,177 metabolites, revealing novel insights. The findings demonstrate that low concentrations of SPB exerted multiple beneficial effects on rice roots, leaves, and the root‐soil system, facilitating rapid cell division and enhancing antioxidant defense mechanisms. These results provide a vital foundation for understanding ATH metabolic pathways and advocating for widespread SPB application, offering significant implications for sustainable land management.

## Graphical abstract

The idea of turning sewage sludge waste into plant biostimulants treasure provides an innovative paradigm for reducing the addition of chemicals in agricultural production. 51 plant hormones and 1,177 metabolites have been detected for the first time, deepening the understanding of sludge alkaline thermal hydrolysis at the molecular level.

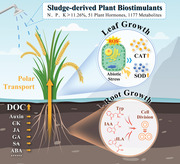

## 1 Introduction

With the acceleration of global urbanization, the amount of domestic sewage discharged by urban residents has reached 360 km^3^ year^−1^.^[^ ^1^ ^]^ Sewage treatment plants, as important urban environmental infrastructure, reduce over 90% of pollutants in sewage before returning it to natural water bodies or for reuse. However, half of these reduced pollutants are transferred to sludge. Globally, 50% of carbon,^[^ ^2^ ^]^ 40% of nitrogen,^[^ ^3^ ^]^ and 90% of phosphorus^[^ ^4^ ^]^ in the wastewater generated by 3.1 billion people^[^ ^5^ ^]^ per day are enriched in sludge. Additionally, sludge contains ≈10^6^ gene mL^−1^ of pathogenic microorganisms,^[^ ^6^ ^]^ ≈10^2^–10^3^ µg (g dry weight)^−1^ of heavy metals,^[^ ^7^ ^]^ and ≈10^2^–10^4^ ng (g dry weight)^−1^ of persistent organic pollutants.^[^ ^8^ ^]^ If sludge is not effectively treated, it can re‐enter the ecosystem as a pollutant‐enriched entity or a secondary source of pollution, significantly compromising the effectiveness of urban infrastructures, such as sewage treatment plants, and causing significant waste of biomass resources. Therefore, sludge management has become a critical factor in ensuring the water quality of urban environments.

Humanity's ancient agricultural civilization has nurtured the traditional production and life mode of “using manure to fertilize fields” and “circularity between humans and land.” Manure has not only not become an environmental burden, but has also become a resource for agricultural development. The traditional agriculture's collection, fermentation, and use of manure not only ensure soil fertility but also address environmental pollution caused by fecal waste and waste materials.^[^ ^8^ ^]^ This has become a symbolic achievement of the concept of traditional agricultural circular economy worldwide. In “The Farmers of Four Thousand Years,” which mainly regarded as the “global organic agriculture bible,” farmers’ practices are described as an integral part of the overall ecological balance, embodying the cycle between humans and the land.^[^ ^9^ ^]^ However, with the accelerating process of industrialization and urbanization, and the completion of urban infrastructure and sewage treatment networks, most urban domestic sewage enters treatment plants through pipelines, disrupting the nutrient cycle chain. Furthermore, the rapid development of the chemical fertilizer industry has led to manure being largely replaced by chemical fertilizers, cutting off the path of returning manure to fields. These two major changes have caused a break in the cycle between humans and the land, leading to a waste of resources and energy and ecological environmental problems.

Utilizing sludge for land applications, such as agricultural fertilizer, land improvement, afforestation of barren areas, nurturing seedlings, and landscaping, is the key strategy to overcome resource and environmental constraints. This strategy reconstructs the cycle of returning domestic sewage to fields, achieving a win‐win situation for both ecosystem and human health. It is an essential approach to effectively address the sludge issue. Drawing from the experiences of developed countries, utilization of sludge in land applications exceeds 50% in countries, such as the UK, France, and Spain,^[^ ^10^ ^]^ reaching 65% in the United States^[^ ^11^ ^]^ and over 75% in Denmark,^[^ ^10b^ ^]^ a leading agricultural nation. Ireland, known as the “European estate,” surpasses 90% utilization.^[^ ^12^ ^]^ These countries have also reached a preliminary consensus that after meeting certain requirements or standards for sludge treatment, land utilization should be prioritized under technical guidance. However, they also emphasize the need for regular monitoring and tracking assessment of pollution risks post‐sludge land utilization, given the complex origins of sludge and the emergence of numerous pollutants.^[^ ^13^ ^]^ Consequently, separating substances beneficial to agricultural production from the complex pollutant system of sludge is the core technical issue in sludge return to land.

In 2021, our team utilized alkaline thermal hydrolysis (ATH) treatment of sludge and discovered two high‐value products, indole‐3‐acetic acid (IAA) and hydroxyphenylacetic acid, in the soluble organic matter (DOM) of the supernatant during solid‐liquid separation.^[^ ^14^ ^]^ This discovery has significantly attracted scholars’ attention in recent years regarding the molecular composition of DOM in alkaline hydrothermal liquids, especially with the application of FT‐ICR‐MS methods deepening the understanding of the full range of organic molecular categories.^[^ ^15^ ^]^ The ATH process accelerates the breakdown of organic floccules in extracellular polymeric substances in sludge, releasing cellular contents and hydrolyzing a large amount of organic matter into the liquid phase. Deamination reactions are the most frequent, leading to the identification of main components in DOMs, including nitrogen‐rich compounds (similar to proteins), amino sugars, lignin‐like groups, and lipids, accompanied by a high proportion of nitrogen‐containing compounds with aromatic and N‐heterocyclic rings (>80% of DOMs).^[^ ^16^ ^]^ From the perspective of producing organic fertilizer from sludge, the DOM environment created by ATH treatment for 12 h enhances plant effects by upregulating starch and sucrose metabolic pathways.^[^ ^17^ ^]^ Furthermore, the “ATH + land utilization” process, compared to traditional sludge treatment methods, possesses significant advantages in terms of reducing carbon emissions throughout the life cycle, with carbon emissions as low as 1080.64 kg CO_2_ (t dry weight)^−1^ and 3.6% of carbon (36.34 kg CO_2_ (t dry weight)^−1^) can be utilized as high‐value resources through carbon sequestration.^[^ ^18^ ^]^ Although these studies have explored high‐value resource utilization, they have not examined specific molecular levels in their component‐based understanding. This is related to the low levels of plant hormones (typically in the ng g^−1^ or even pg g^−1^ range), poor environmental stability (requiring high operating conditions), the presence of numerous interferents in the matrix (requiring crucial pretreatment), and difficulties in obtaining standard substances. Another critical point is that environmental researchers concentrating on sludge mainly prioritize pollution reduction. While they may employ resource‐based methods for sludge treatment, their research frequently halts the production of specific resource materials. For instance, they may identify beneficial compounds, such as indoleacetic acid or hydroxyphenylacetic acid, that promote plant growth following ATH treatment of sludge, without considering the broader implications or further applications of these findings. However, the raw materials for producing these substances are complex,^[^ ^19^ ^]^ and these high‐value products are not present in a single pure form in sludge products. Moreover, the detected indoleacetic acid and hydroxyphenylacetic acid are present only in trace amounts. With the development of novel sludge treatment technologies, such as ATH, there has been a growing interest in extracting valuable compounds from sludge for beneficial applications. ATH treatment accelerates the breakdown of organic matter in sludge, yielding supernatant rich in soluble organic compounds, including IAA and hydroxyphenylacetic acid. Recent studies have highlighted the agricultural potential of these compounds, sparking further investigation into the molecular composition and agricultural implications of sludge‐derived plant biostimulants (SPB) produced through ATH. Therefore, it is necessary to have a deeper understanding of the material composition of ATH products, followed by a lack of understanding of the mechanism of the impacts on roots, leaves, soil, etc., from the perspective of the entire crop growth cycle after applying ATH products to crops.

With the acceleration of global urbanization, the amount of domestic sewage discharged by urban residents has reached 360 km^3^ year⁻¹. Sewage treatment plants, as crucial urban environmental infrastructure, mitigate over 90% of pollutants in sewage before discharging it into natural water bodies or reusing it. However, half of these reduced pollutants are transferred to sludge, containing significant amounts of carbon, nitrogen, and phosphorus, as well as pathogenic microorganisms, heavy metals, and persistent organic pollutants. If not effectively treated, sludge can become a pollutant‐enriched entity, undermining urban infrastructure, and wasting biomass resources. Therefore, managing sludge is pivotal for maintaining urban water quality. Recent research has highlighted the potential of SPB for sustainable agriculture, promoting nutrient uptake, root development, and stress tolerance in plants. ATH is an advanced sludge treatment method that decomposes organic matter under high temperature and alkaline conditions, producing valuable by‐products, such as IAA and hydroxyphenylacetic acid, which can enhance soil fertility and plant growth. Despite these advancements, significant gaps remain in understanding the molecular‐level composition of SPBs and their long‐term impacts on crops and soil health.

This study aimed to address critical research gaps concerning the molecular‐level composition of SPB and their comprehensive impact on crop growth. One of the primary objectives is to deepen understanding of the material composition of SPB at a molecular level through non‐targeted and targeted metabolomics analyses. This included leveraging targeted metabolomics for quantitative analysis and utilizing a self‐constructed database of standard substances to enhance qualitative analysis. Furthermore, the response patterns and mechanisms of various factors were assessed, including roots, leaves, and soil, throughout the entire growth cycle of rice under conditions where SPB was used as a substitute for chemical fertilizers. By bridging these gaps in knowledge, the findings may provide insights into the molecular composition of SPB and elucidate their comprehensive impacts on crop growth, considering sustainability and ecological implications in agricultural practices.

## 2 Results and Discussion

### 2.1 Decoding Sludge‐Derived Plant Biostimulants from a Molecular Level

#### 2.1.1 Physiochemical and Security Properties

The organic matter in sludge contains 60% protein,^[^ ^20^ ^]^ making it a typical high‐nitrogen biomass. The ATH process accelerates the decomposition of sludge cells and the degradation of organic matter, particularly enhancing the dissolution efficiency of proteins.^[^ ^14^ ^]^ ATH enhances the dissolution efficiency of proteins through a combination of high temperature and alkaline conditions. These conditions disrupt the cellular structure of the sludge, leading to the breakdown of cell walls and membranes. The high temperatures facilitate the hydrolysis of complex organic molecules, including proteins, into smaller, more soluble fragments. Simultaneously, the alkaline environment promotes the ionization of amino acids and proteins, increasing their solubility in water. This process not only accelerates the release of organic nitrogen from microbial extracellular polymeric substances but also inactivates microbes, further contributing to the release of nitrogen‐containing substances from cells. Additionally, the accumulation of negative charges on cell surfaces under alkaline conditions induces the deaggregation and dissolution of extracellular organic nitrogen due to high electrostatic repulsion, further enhancing the dissolution efficiency of proteins. SPB retains abundant nitrogen resources, with a total nitrogen content of 4598 ± 129 mg L^−1^ (**Table** **1**), including soluble humic acids, soluble proteins, free amino acids, soluble ammonia nitrogen, and other protein hydrolysates. This is closely related to the hydrothermal and alkaline treatment conditions. This is strongly influenced by the conditions of hydrothermal and alkaline treatment. Hydrothermal treatment enhances the release of organic nitrogen from microbial extracellular polymeric substances,^[^ ^21^ ^]^ while the high‐temperature environment causes nitrogen‐containing substances to be released from cells as some microbes are inactivated. Moreover, under alkaline conditions, the accumulation of negative charges on cell surfaces leads to the deaggregation and dissolution of extracellular organic nitrogen due to high electrostatic repulsion.^[^ ^21^ ^]^ Under high pH conditions, certain cells lost their ability to maintain adequate turgor pressure, leading to cell inactivation, structural damage, and subsequent nitrogen release. It is noteworthy that ATH treatment is mainly accompanied by the hydrolysis of a large amount of macromolecular organic matter into smaller molecules, as evidenced by the weight‐average molecular weight showing that the proportion of 0–3 kDa reaches a maximum of 44.84%. Ammonia is the mineralization product of most organic nitrogen, and ammonia nitrogen accounted for only 25.60% of total nitrogen in the present study. In our previous study,^[^ ^14^ ^]^ we found that in systems using CaO as an alkali source, the bridging and chelating effects of Ca^2+^ with amino acids effectively maintain high retention of nitrogen in organic matter, thereby increasing nitrogen dissolution while reducing conversion to ammonia nitrogen, ensuring an adequate supply of precursor materials for the production of IAA. Similar results have been confirmed, in which amine‐N, amino‐N, and heterocyclic‐N (i.e., pyridine‐N, and quaternary‐N) have been found as predominant nitrogen species in DOMs, significantly higher than inorganic‐N content.^[^ ^15^ ^]^ With the increase in pH (from 9 to 12), the concentration of amine‐N sharply decreases, accompanied by a significant increase in amino‐N concentration. Glycine, alanine, glutamic acid, phenylalanine, and leucine are the most abundant free amino acids in SPB.^[^ ^16^ ^]^ In addition, the enriched N, P, and K element contents in SPB (> 11.26%, dry weight) are significantly higher than the industrial standard of agricultural organic fertilizer (4%, N + P_2_O_5_ + K_2_O, dry weight),^[^ ^22^ ^]^ providing remarkable nutrient supply for crop growth and maintaining soil nutrient balance. Furthermore, the cumulative effect of SPB over successive cropping seasons poses challenges for sustainable rice production and necessitates proactive management strategies. While seed germination and root elongation are widely recognized for their sensitivity to a range of contaminants and provide early warnings of phytotoxicity, they primarily assess acute toxicity and might miss chronic or sub‐lethal effects. Although earthworm acute toxicity tests provide valuable insights into soil health and potential impacts on soil biota, mortality alone may not reflect sub‐lethal and reproductive effects, which are critical for understanding long‐term ecological impacts. Therefore, incorporating chronic toxicity tests and additional sensitive bioassays is suggested for a more thorough evaluation of SPB safety. Understanding the dynamics of SPB infection and its management is essential for mitigating yield losses and ensuring the long‐term sustainability of rice production in affected regions.

**Table 1:** Physiochemical and security properties of raw sludge and sludge‐derived plant biostimulants.

Physiochemical Properties		Raw Sludge	SPB
**Basic Quality**	pH	6.59 ± 0.03	11.29 ± 0.04
	Electrical Conductivity (μ s cm^−1^)	0.77 ± 0.24	5.14 ± 0.37
	Total Solid (%)	18.97 ± 0.13	–
	Volatile Solids/Total Solid (%)	32.69 ± 0.05	–
	Total Nitrogen	4.89 ± 0.29 [% TS]	4,598 ± 129 [mg L^−1^]
	Soluble Proteins (mg L^−1^)	469.1 ± 24.0	20,565 ± 811
	Soluble Humic Acids (mg L^−1^)	125.3 ± 19.7	1,086.3 ± 30.4
	Free Amino Acids (g L^−1^)	–	131.8 ± 5.00
	Soluble Ammonia Nitrogen	574.3 ± 13.9	1,177 ± 49
**Weight‐average Molecular Weight (Mw) Distributions**	0∼3 kDa	–	44.84%
	3∼10 kDa	–	11.82%
	10∼30 kDa	–	11.79%
	30∼100 kDa	–	10.39%
	100∼300 kDa	–	7.98%
	300∼1,000 kDa	–	7.93%
	>1,000 kDa	–	5.24%

Security Properties		Raw Sludge	SPB	
	Ca (g (kg‐TS)^−1^)	27.2 ± 2.6	3.80 ± 0.15	
**Polyvalent Metals**	Mg (mg (kg‐TS)^−1^)	5,457 ± 85.0	1.20 ± 0.05	
	Fe (mg (kg‐TS)^−1^)	446 ± 19.7	2.50 ± 0.20	
	Al (mg (kg‐TS)^−1^)	1,550 ± 19.7	34.0 ± 1.4	
	Cu (mg (kg‐TS)^−1^)	108.34 ± 2.35	0.43 ± 0.03	
	Zn (mg (kg‐TS)^−1^)	229.35 ± 12.9	4.13 ± 0.05	
	Ni (mg (kg‐TS)^−1^)	65.41 ± 2.37	7.98 ± 0.09	
	Mn (mg (kg‐TS)^−1^)	195.21 ± 13.05	17.7 ± 0.2	
**Harmful Metals**	Cr (mg (kg‐TS)^−1^)	29.03 ± 0.21	0.24 ± 0.00	
	As (mg (kg‐TS)^−1^)	33.12 ± 0.16	0.30 ± 0.02	
	Cd (mg (kg‐TS)^−1^)	0.82 ± 0.14	0.14 ± 0.01	
	Pb (mg (kg‐TS)^−1^)	N.D.	N.D.	
	Hg (mg (kg‐TS)^−1^)	N.D.	N.D.	
**Earthworm Acute Toxicity Test Death Rate**	Exposure Concentration	–	7 d	14 d
	0 mg kg^−1^	–	0	0
	20.6 mg kg^−1^	–	0	0
	61.7 mg kg^−1^	–	2.5%	2.5%
	185 mg kg^−1^	–	5%	10%
	556 mg kg^−1^	–	7.5%	12.5%
	1,667 mg kg^−1^	–	10%	15%
	5,000 mg kg^−1^	–	15	20

SPB, Sludge‐derived plant biostimulants.

**Figure 1:**
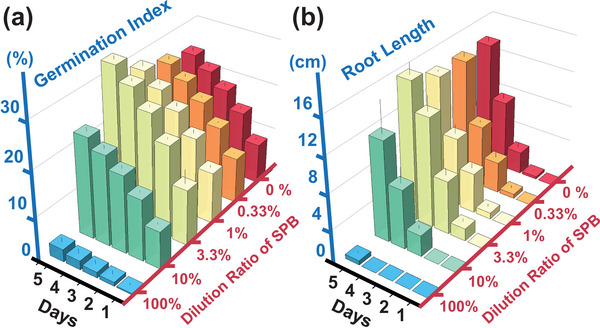
The response of a) germination index and b) root length to sludge‐derived plant biostimulants application. A single‐factor controlled variable experiment was conducted using SPB concentration, with one blank group (containing only distilled water) and five experimental groups (0.33%, 1%, 3.3%, 10%, 100%). Three replicates were set up for each group. The petri dishes were incubated in a light‐avoiding germination chamber at 25 ± 1 °C for 5 days.

Considering security properties, the metal content in SPB is significantly lower compared to the original sludge, which may be related to the separation of insoluble salts formed by metal ions during centrifugation. Particularly, the harmful metal content is significantly lower than the standards set by multiple countries for organic fertilizer products. Seed germination and root elongation are critical and sensitive periods during the rice growth cycle. Therefore, seed germination is a commonly used method for testing plant toxicity. As illustrated in **Figure** **1**, under conditions of SPB concentrations ranging from 0.33% to 3.3%, there was a positive promotion effect on rice seed germination and root elongation, with the promotion benefits increasing with concentration. However, when the SPB concentration was further elevated by 10% or even 100%, a significant inhibitory effect was observed. This effect was closely associated with the increased concentrations of specific substances in SPB exceeding the tolerance range of rice seeds. This suggests that SPB should be applied at appropriate concentrations to ensure non‐toxicity and significant promotion effects on seed germination and root elongation. Furthermore, as presented in Table 1, the lethal concentration 50 (LC50) of SPB for earthworms was higher than 5000 mg kg^−1^, while in actual applications, the concentration of SPB was significantly lower than 5000 mg kg^−1^ (<10 mg kg^−1^). Therefore, from the perspective of earthworm toxicity, SPB can be considered safe for agricultural applications. For future studies, we recommend the inclusion of a diverse range of bioassays to capture potential toxic effects more comprehensively. These include microbial assays (e.g., Microtox test) for detecting acute toxicity at low contaminant concentrations, aquatic toxicity tests with organisms like Daphnia magna and fish embryos, and higher plant assays to assess endpoints such as photosynthetic efficiency and chlorophyll content. Additionally, long‐term exposure studies on organisms, detailed chemical characterization of SPB, and advanced omics approaches (genomics, proteomics, and metabolomics) should be utilized to provide deeper insights into the molecular mechanisms of toxicity and identify biomarkers of exposure and effect. Comparing SPB characteristics with established safety standards for organic fertilizers and biostimulants in different countries will further ensure compliance and safety.

#### 2.1.2 Identification of Plant Hormones and Functional Metabolites

In the present study, we identified and classified a variety of metabolites, each playing distinct roles in plant physiology. Below is a summary of the key metabolites detected and their specific physiological functions:

Primary metabolites, such as sugars, amino acids, and organic acids are vital for the fundamental processes of plant life. Glucose and fructose are essential for energy production through cellular respiration, serving as building blocks for more complex carbohydrates crucial for plant growth and development. Sucrose functions as the main transport form of sugar in plants, moving from the photosynthetic tissues (source) to non‐photosynthetic tissues (sink) where it is utilized or stored. Amino acids, including glutamine and glutamate are involved in nitrogen assimilation and transport, acting as key intermediates in the synthesis of other amino acids and nitrogenous compounds. Proline accumulates in response to osmotic stress, functioning as an osmoprotectant that helps stabilize proteins and membranes under stress conditions. Organic acids, including citric acid and malic acid, are integral components of the citric acid cycle (Krebs cycle), pivotal for energy production, and play roles in pH regulation and metal ion chelation within plant cells.

Secondary metabolites, such as alkaloids, phenolics, and terpenoids, play significant roles in plant defense and adaptation. Nicotine, an alkaloid, acts as a defense compound against herbivores and influences plant signaling pathways involved in growth and stress responses. Phenolics, such as flavonoids contribute to UV protection by absorbing harmful radiation, aid in flower coloration for pollination, and defend against pathogens through their antioxidant properties. Lignin provides structural support by reinforcing cell walls and contributes to water transport efficiency and resistance to pathogen invasion. Terpenoids, such as menthol, found in peppermint, play roles in plant defense by deterring herbivores and attracting pollinators through their aromatic properties. Carotenoids are involved in photosynthesis by protecting chlorophyll from photodamage and act as precursors to signaling molecules, such as abscisic acid, important for stress responses. Each class of metabolites is crucial for maintaining plant health, growth, and adaptation to environmental challenges, highlighting the complex biochemical networks that sustain plant life and resilience.

In this study, a highly sensitive platform (AB Sciex QTRAP 6500 LC‐MS/MS platform) and a self‐built reference database were utilized to quantitatively detect 88 types of free and bound plant hormones belonging to eight major classes (abscisic acid, auxins, cytokinins, gibberellins, jasmonic acid, salicylic acid, brassinosteroids, and ethylene) in SPB innovately. A total of 51 plant hormones were identified from the SPB samples (**Table** **2**), including 17 auxins (32479.2 ng mL^−1^), 16 cytokinins (CK, 148.9 ng mL^−1^), 6 jasmonic acids (JA, 93.0 ng mL^−1^), 5 gibberellins (GA, 49.3 ng mL^−1^), 4 salicylic acids (SA, 11023.9 ng mL^−1^), 2 abscisic acids (ABA, 86.5 ng mL^−1^), and 1 ethylene (ETH, 200.1 ng mL^−1^). Among them, L‐tryptophan (L‐Trp, 25849.6 ng mL^−1^), L‐phenylalanine (10834.2 ng mL^−1^), indole (3671.0 ng mL^−1^), indole‐3‐lactic acid (ILA, 1428.3 ng mL^−1^), IAA (682.0 ng mL^−1^), indole‐3‐acetaldehyde (505.0 ng mL^−1^), 1‐aminocyclopropane‐1‐carboxylic acid (200.1 ng mL^−1^), indole‐3‐acetate (188.1 ng mL^−1^), cinnamic acid (141.5 ng mL^−1^), and 3‐indolepropionic acid (91.8 ng mL^−1^) were the top 10 plant hormones in terms of detected concentrations. This is the first study to report on the preparation and detection of composite plant hormone products derived from waste samples.

**Table 2:** Plant hormone concentrations of sludge‐derived plant biostimulants.

Class	Compounds	Formula	Concentrations [ng mL^−1^]	Class	Compounds	Formula	Concentrations [ng mL^−1^]	Class	Compounds	Formula	Concentrations [ng mL^−1^]
**Auxin**	L‐tryptophan	C_11_H_12_N_2_O_2_	25849.6	**CK**	2‐Methylthio‐cis‐zeatin riboside	C_16_H_23_N_5_O_5_S	30.9	**JA**	12‐Hydroxyjasmonic acid	C_12_H_18_O_4_	54.4
	Indole	C_8_H_7_N	3671.0		2‐Methylthio‐N6‐isopentenyladenosine	C_16_H_23_N_5_O_4_S	28.4		cis(+)‐12‐Oxophytodienoic acid	C_18_H_28_O_3_	24.8
	Indole‐3‐lactic acid	C_11_H_11_NO_3_	1428.3		2‐Methylthio‐N6‐isopentenyladenine	C_11_H_15_N_5_S	22.6		Dihydrojasmonic acid	C_12_H_20_O_3_	9.6
	Indole‐3‐acetic acid	C_10_H_9_NO_2_	682.0		N6‐isopentenyladenosine	C_15_H_21_N_5_O_4_	12.9		Jasmonic acid	C_12_H_18_O_3_	1.9
	Indole‐3‐carboxaldehyde	C_9_H_7_NO	505.0		N6‐Isopentenyl‐adenine‐7‐glucoside	C_16_H_23_N_5_O_5_	10.6		Jasmonate‐1‐aminocyclopropane‐1‐carboxylic acid	C_16_H_22_NO_4_	1.7
	Indole‐3‐carboxylic acid	C_9_H_7_NO_2_	188.1		cis‐Zeatin	C_10_H_13_N_5_O	9.2		Jasmonoyl‐L‐isoleucine	C_18_H_29_NO_4_	0.6
	3‐Indolepropionic acid	C_11_H_11_NO_2_	91.8		N6‐Isopentenyl‐adenine‐9‐glucoside	C_16_H_23_N_5_O_5_	7.0	**GA**	Gibberellin A29	C_19_H_24_O_6_	41.6
	N‐(3‐Indolylacetyl)‐L‐alanine	C_13_H_14_N_2_O_3_	19.5		N6‐isopentenyladenine	C_10_H_13_N_5_	6.3		Gibberellin A1	C_19_H_24_O_6_	6.2
	N‐(3‐Indolylacetyl)‐L‐leucine	C_16_H_20_N_2_O_3_	13.0		Dihydrozeatin	C_10_H_15_N_5_O	5.1		Gibberellin A12 aldehyde	C_20_H_28_O_3_	0.7
	Tryptamine	C_10_H_12_N_2_	12.1		para‐Topolin riboside	C_17_H_19_N_5_O_5_	5.0		Gibberellin A7	C_19_H_22_O_5_	0.4
	N‐(3‐Indolylacetyl)‐L‐phenylalanine	C_19_H_18_N_2_O_3_	6.0		2‐Methylthio‐cis‐zeatin	C_11_H_15_N_5_OS	4.1		Gibberellin A15	C_20_H_26_O_4_	0.4
	N‐(3‐Indolylacetyl)‐L‐valine	C_15_H_18_N_2_O_3_	5.2		cis‐Zeatin riboside	C_15_H_21_N_5_O_5_	2.2	**SA**	L‐Phenylalanine	C_9_H_11_NO_2_	10834.2
	Indole‐3‐acetyl glycine	C_12_H_12_N_2_O_3_	3.0		Dihydrozeatin‐O‐glucoside riboside	C_21_H_33_N_5_O_10_	1.9		trans‐Cinnamic acid	C_9_H_8_O_2_	141.5
	Indole‐3‐acetyl‐L‐tryptophan	C_21_H_19_N_3_O_3_	2.8		Kinetin	C_10_H_9_N_5_O	1.3		Salicylic acid	C_7_H_6_O_3_	37.6
	3‐Indole acetamide	C_10_H_10_N_2_O	1.5		trans‐Zeatin riboside	C_15_H_21_N_5_O_5_	0.7		Salicylic acid 2‐O‐β‐glucoside	C_13_H_16_O_8_	10.6
	Indole‐3‐butyric acid	C_12_H_13_NO_2_	0.2		Kinetin riboside	C_15_H_17_N_5_O_5_	0.7	**ABA**	Abscisic aldehyde	C_15_H_20_O_3_	82.3
	Methyl indole‐3‐acetate	C_11_H_11_NO_2_	0.1	**ETH**	1‐Aminocyclopropanecarboxylic acid	C_4_H_7_NO_2_	200.1		Abscisic acid	C_15_H_20_O_4_	4.2

ABA, Abscisic Acid; CK, Cytokinins; ETH, Ethylene; GA, Gibberellins; JA, Jasmonates; SA, Salicylates.

Besides plant hormones, we leveraged the advantages of non‐targeted metabolomics for the qualitative analysis and targeted metabolomics for the quantitative analysis to establish a secondary spectrum information based on a self‐built target standard database, involving 1800 primary metabolites and 28 000 secondary metabolites, to examine the hydrolysates of SPB. A total of 1177 metabolites were detected (Figure S1, Supporting Information), with amino acids and their derivatives being the most abundant at 34.02%. This content has some comparative value with protein/peptides (1 – 85%) and free amino acids (2 – 18%) found in animal and plant protein hydrolysates,^[^ ^23^ ^]^ indicating that specific peptides can serve as messenger molecules for plant defense, growth, and development.^[^ ^24^ ^]^ Additionally, other biologically stimulating compounds were found in the SPB hydrolysates: Alkaloids (12.35%), which are nitrogen‐containing natural organic compounds, mostly nitrogen‐containing heterocyclic compounds, play important roles in plants’ response to environmental stress, pest defense, and are effective insecticides in agriculture. They are also effective components of several medicinal plants with functions, such as anti‐tumor, anti‐inflammatory, immune enhancement, and cardiovascular disease treatment.^[^ ^25^ ^]^ Lipids (8.46%) include fatty acids and their naturally occurring derivatives (e.g., esters or amines), as well as compounds related to their biosynthesis and functions, playing essential roles in plant growth and development, energy conversion, material transport, information transmission, and metabolic regulation.^[^ ^26^ ^]^ Terpenoids (5.87%) are a collective term for isoprene polymers and their derivatives, possessing functions, such as antimicrobial, insecticidal, and antifungal properties, as well as pheromonal effects. They are important components of herbicides and insecticides, and they have medicinal properties, such as anti‐inflammatory and anti‐tumor activities.^[^ ^27^ ^]^ This study, leveraging the advantages of a self‐built target standard database, concentrated on the specific molecular level of SPB, providing a crucial foundation for metabolic analysis of ATH processes and the high‐value transformation of sludge.

### 2.2 Rice Response to Sludge‐Derived Plant Biostimulants

#### 2.2.1 Rice Root Growth Patterns During the Seedling Stage

**Figure 2:**
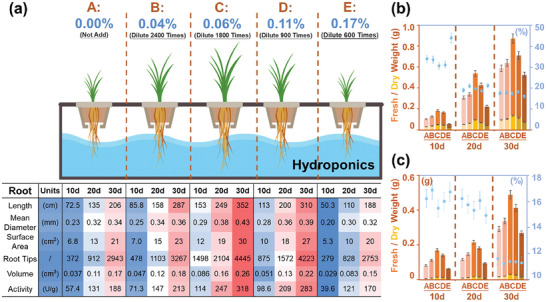
The response of rice to sludge‐derived plant biostimulants (SPB) in hydroponic experiments: a) the root system architecture of rice seedlings, the weight and the proportion of b) aboveground and c) belowground biomass. SPB concentration was used as a single‐factor controlled variable. One blank group (containing only distilled water) and four experimental groups (0.04%, 0.06%, 0.11%, and 0.17%) were set up. Each group had four replicates, with six rice seedlings in each replicate. The light intensity was set at 300–320 µmol·(m^2^·s)^−1^, relative humidity ranged from 65% to 80%, and the photoperiod consisted of 16 h of light, followed by 8 h of darkness. The temperature was maintained at 22 °C. The seedlings were cultured continuously for 30 days, and the nutrient solution changed every 2 days. Each experimental condition shown in the figure was performed with three biological replicates.

The ability of rice to obtain water and nutrients largely depends on the structure of its root system. Therefore, the growth of rice roots directly affects the overall growth, development, nutritional value, and yield of rice.^[^ ^28^ ^]^ The effect of SPB on rice yield over the crop's life cycle was found to be significant, with notable implications for agricultural practices and policy‐making. Throughout the rice plant's growth stages, SPB could inflict substantial damage, from stunted seedling growth to compromised photosynthesis and nutrient uptake during maturity. The application of different concentrations of SPB has a significant impact on the root system architecture of rice seedlings (**Figure** **2**a). From days 10 to 30, the total root length, root surface area, root diameter, root volume, and root tip number of rice roots in groups B–D treated with 0.04 to 0.11% concentrations of SPB were all elevated (*p* < 0.05). Among them, the optimal enhancement was found at a concentration of 0.06%. Measurements on day 10 exhibited respective increases of 153.15 cm in length, 11.91 cm^2^ in area, 0.29 cm in diameter, 0.09 cm^3^ in volume, and 1498 roots. These represented increases of 111.16%, 76.03%, 25.03%, 133.33%, and 302.60% compared with the control group. These results indicate that a certain concentration range of SPB contributes to promoting the root growth of rice seedlings. This is because SPB contains multiple auxins, which are key regulatory factors for lateral root development.^[^ ^29^ ^]^ Particularly, the morphological characteristics of roots are controlled by the level and distribution of IAA,^[^ ^30^ ^]^ which promotes the growth and development of rice roots. The IAA content in SPB in this study was 682.0 ng mL^−1^, making it one of the main components of plant hormones. The appropriate concentration of IAA is necessary for establishing a rapidly dividing initial cell group,^[^ ^29^ ^]^ indicating the significant growth advantage of the root system in groups B ≈ D at day 10. Notably, two growth substances found in high concentrations in SPB, L‐Trp (25849.6 ng mL^−1^) and ILA (1428.3 ng mL^−1^), can serve as precursors for the IAA biosynthesis pathway. They are converted into IAA when needed, thereby promoting root growth and expansion.^[^ ^31^ ^]^ ILA can regulate the formation of root hairs, explaining the significant increase in root tip number in groups B–D. Additionally, the polar transport of hormones in roots, where IAA moves to the root tips and the junction between the root tip and root crown, is necessary for lateral root development.^[^ ^32^ ^]^ The addition of exogenous hormones may enhance this polar transport, leading to increased root volume and number. Apart from plant hormones, the well‐developed root system of rice seedlings in groups B–D also benefits from the addition of appropriate nitrogen and phosphorus nutrients. Phosphorus is mainly considered as the most limiting essential nutrient, and the acquisition of plant phosphorus largely depends on root exploration. The phosphorus element in SPB provides possibilities for synthesizing essential components, such as energy molecules (DNA, RNA, and ATP) and membrane phospholipids. Furthermore, the increased rate of root elongation and deeper root system may accelerate the absorption rate of nutrients in rice,^[^ ^33^ ^]^ forming a beneficial cycle. Studies have reported that large and deep root systems are the basis for high nitrogen utilization efficiency.^[^ ^34^ ^]^

However, when a concentration of 0.17% SPB was added, the growth of rice roots was significantly inhibited, especially in the early stages of growth. On day 10, the total root length, root surface area, root diameter, root volume, and root tip number of rice roots decreased by 30.64%, 21.72%, 11.89%, 20.72%, and 25.09%, respectively, compared with the control group. High concentrations of SPB exhibited a certain inhibitory effect on the root system of rice seedlings. On one hand, this could be related to the primary form of nitrogen nutrients in SPB, which is predominantly NO_3_^−^. The application of nitrogen nutrients can regulate the carbon‐nitrogen metabolism and carbohydrate transport distribution inside plants, thereby exerting favorable or unfavorable effects on root development. Nitrate nitrogen is one of the most mobile mineral nutrient ions, and it can easily enter the rhizosphere soil with the permeation of the extracting solution.^[^ ^33^ ^]^ According to the theory of “dual regulation pathway of root growth induced by a local signal of NO_3_^−^ and systemic nutrient inhibition”^[^ ^35^ ^]^ trace amounts of NO_3_^−^ can stimulate root growth as a signaling molecule. However, at higher concentrations, the role of NO_3_^−^ as a nutrient far exceeds its signaling function, thereby inhibiting root growth. On the other hand, excessive growth hormone compounds can inhibit root growth, such as IAA exhibiting phytotoxicity at high concentrations.^[^ ^31c^ ^]^ Prior research also indicated that L‐Trp concentrations exceeding 10^−5^ m (equivalent to 2.04 ng mL^−1^) could have inhibitory effects of 10% to 30% on seedling root growth, with specific degrees varying depending on the plant type.^[^ ^31c^ ^]^ In this study, the external L‐Trp concentration in group E was ≈40 ng mL^−1^, significantly exceeding the inhibitory concentration. Although the concentrations of L‐Trp in groups B–D were also higher than the theoretical inhibitory concentration, some of the L‐Trp can be converted into positive factors that promote development, such as IAA and indole‐3‐ethanol, thereby weakening its negative effects.

Plant root vitality is an important factor reflecting root growth and has significant implications for plant development.^[^ ^36^ ^]^ After 10 days of hydroponic cultivation of rice seedlings, compared to the control group, the root vitality of groups B to D increased significantly by 24.14%, 98.09%, and 71.64%, respectively, while group E showed a significant decrease in root vitality by 31.04%. Across the three time periods, rice seedling root vitality reached its maximum value when treated with 0.06% concentration of SPB, and there were significant differences compared with other groups. The application of appropriate concentrations (0.04–0.11%) of SPB significantly promoted the root vitality of hydroponically grown rice seedlings at different time periods, contributing to root growth and development. Besides the stimulating effect of plant hormones, this could also be related to the abundant presence of Ca^2+^ in the extract from sludge after alkaline stabilization. Exogenous calcium can enhance plant root vitality and stress resistance by strengthening the plant's antioxidant enzyme system, reducing membrane lipid peroxidation, and employing other physiological regulatory mechanisms.^[^ ^37^ ^]^ Additionally, the nitrogen in SPB also contributes to the formation of a well‐structured root system in rice seedlings, enhancing root vitality and promoting root growth.

Over time, the impact of SPB on rice seedlings appeared to gradually diminish. On day 30, the levels of indicators in group C increased by 71.27%, 39.85%, 26.47%, 44.44%, and 51.01%, respectively, compared with the control group, while in group E, they decreased by 8.56%, 7.29%, 7.59%, 14.23%, and 6.48% respectively. The proportion of aboveground (Figure 2b) and belowground (Figure 2c) biomass to the total mass of rice plants provides more visual evidence to confirm this conclusion. Compared with the results on day 10, the differences between the promoting and inhibiting groups were narrowed. This could be attributed to the gradual decrease in the content of plant hormones utilized by rice seedlings, leading to the weakening of their impact. Additionally, this is related to the greater sensitivity and faster division of early‐stage cells to plant hormones. Under the influence of auxins, the root system initially establishes a group of active, rapidly dividing cells, gradually differentiating into lateral root primordia.^[^ ^29^ ^]^

#### 2.2.2 Rice Leaf Quality During the Seedling Stage

Strong root systems promote photosynthesis in the aboveground parts, while an adequate supply of photosynthetic products provides essential water and nutrients for root growth, making leaf growth and development crucial.^[^ ^38^ ^]^ Under normal conditions, plants have an inherent reactive oxygen species (ROS) scavenging system that maintains a relative balance in enzyme activities within the leaves.^[^ ^39^ ^]^ Therefore, measuring enzyme activity in rice seedling leaves is relevant for studying the quality of rice grains after the application of extracts. The research results showed significant differences in enzyme activity among groups A to E (*p* < 0.05). At 30 days, the activities of catalase (CAT) in groups A to E were 11.35, 12.70, 16.12, 14.31, and 10.11 U (g·min)^−1^, respectively, while the activities of superoxide dismutase (SOD) were 72.13, 74.92, 83.64, 77.90, and 71.22 U g^−1^, respectively. Compared with the control group, the SOD and CAT activities in the leaf blades of rice seedlings in groups C and D were significantly elevated, with C showing increases of 15.96% and 42.02%, and D showing increases of 8.00% and 26.08%, respectively. The presence of Ca^2+^ in SPB contributes to maintaining the activity of these two antioxidant protection enzymes in plants. This indicates that an appropriate concentration of SPB, especially at a concentration of 0.06%, can enhance plants' ability to withstand environmental stress, effectively defend against reactive oxygen species and other free radicals attacking plant membrane lipids, maintain internal balance in plants, and keep cells at normal metabolic activity levels.^[^ ^39^ ^]^ Additionally, the influence of the extract on CAT and SOD enzyme activities varies across different time periods. This is because rice seedlings have varying tolerance to nutrients, such as Ca^2+^ or nitrogen and phosphorus in active sludge extracts at different stages of growth and development.

In the plant's antioxidant system, reduced glutathione (GSH) is an important non‐enzymatic antioxidant that plays a key role in balancing oxidative and reductive activities within the plant.^[^ ^40^ ^]^ The trend in GSH content changes is similar to the changes in SOD and CAT enzyme activities. Compared to group A, the GSH content in group C significantly increased by 40.45%, 39.55%, and 33.26% at 10, 20, and 30 days, respectively. This also confirms that the application of SPB at concentrations of 0.06% to 0.11% can enhance rice's ability to resist oxidative stress. However, when the SPB concentration reaches 0.17%, it exhibits significant inhibitory effects on SOD and CAT enzyme activities in rice seedlings. This may be related to the lower alkalinity of the nutrient solution when applying low‐concentration active sludge extract and the medium to high alkalinity of the nutrient solution when applying high‐concentration extract. Additionally, high concentrations of SPB are more likely to stimulate the formation of malondialdehyde (MDA). Group E showed a significant increase in rice whole‐plant MDA content by 19.8% and 17.8% compared to group A at 20 and 30 days, respectively, indicating significant differences. MDA is a major product of membrane lipid peroxidation, and its level changes to some extent to reflect the degree of plant damage under stress. High alkalinity stress leads to reduced seedling activity, which further confirms that excessively high concentrations of the extract can hinder plant growth, ultimately leading to yellowing of new leaves, wilting at the top, and root damage.^[^ ^41^ ^]^ When considering root system architecture, root vitality, and leaf enzyme activity, seedlings in group C exhibited the highest performance, suggesting that a concentration of 0.06% is optimal for application. However, the concentration of 0.17% typically has inhibitory effects, indicating it to be an excessive concentration of exogenous additives.

### 2.3 Soil Response to Sludge‐Derived Plant Biostimulants

#### 2.3.1 Physicochemical Properties of Rhizosphere Soil

**Figure 3:**
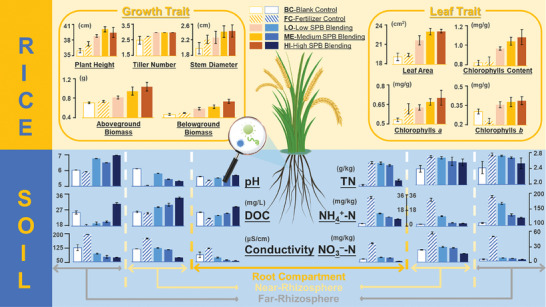
The response of a) soil and b) rice to sludge‐derived plant biostimulants (SPB) in root box experiments. Five treatment groups were set up, including blank control (no fertilizer application), fertilizer control (fertilizer application only), low SPB blending (30% N reduction by fertilizer + 2 mL of SPB), medium SPB blending (30% N reduction by fertilizer + 4 mL of SPB), and high SPB blending (40 mL of SPB only as N source + phosphorus and potash). Urea, potassium dihydrogen phosphate, and potassium chloride were used in the trials as the sources of nitrogen, phosphorus, and potassium in the fertilizers, respectively. Three parallel sets of each test group were set up. The greenhouse was naturally lit and set at 25 ± 1 °C and 8% to 14% humidity for 30 d of continuous incubation.

Applying chemical fertilizers or combining them with SPB can influence the physicochemical properties of the rhizosphere soil, and SPB application optimizes soil properties. As illustrated in **Figure** **3**a, the soil pH in the rhizosphere, near rhizosphere, and far rhizosphere in the control and blank control (BC) groups were 5.67, 6.11, and 6.03, respectively. Adding nitrogen fertilizers to the soil releases a large number of protons through urea ammonification and nitrification, reducing the soil pH. It is evident that the fertilizer control (FC) group exhibited a significant decrease in pH in all three areas, with values of 5.37, 5.05, and 5.82, respectively. However, compared with the fertilizer application group, the SPB combination group showed a significant increase in soil pH, with the highest increase reaching 14.97%. This is partly because of the alkalinity of SPB itself (11.29 ± 0.04), directly increasing soil pH, and partly because the organic extract can enhance the activities of nitrogen‐transforming enzymes, improve nitrogen utilization efficiency, reduce soil nitrate content, and increase soil pH.^[^ ^42^ ^]^ Additionally, the plant hormones present in SPB can alter soil properties, benefiting plant growth. ABA and JA, being acidic themselves, enhance nutrient solubility in the soil.^[^ ^43^ ^]^ SA and CK convert phosphorus into an absorbable form in the soil, promoting seedling root growth.^[^ ^44^ ^]^ As SPB application increases plant nutrient ion utilization efficiency while reducing the input of readily available nutrients, the soil electrical conductivity in all regions significantly decreased compared with conventional fertilization, with reductions ranging from 28.77 to 66.27%. This result aligns with conclusions from experiments using organic water‐soluble fertilizers, further indicating that SPB can reduce soil salinity and mitigate the risk of soil salinization.^[^ ^45^ ^]^

The noteworthy point is that the low, medium, and high SPB blending (LO, ME, and HI) groups showed a significant increase in dissolved organic carbon (DOC) content in all regions, with increases ranging from 8.29% to 68.03%. The higher soil DOC content promotes the ability of plants to utilize carbohydrates, amino acids, polymers, and other substances. Therefore, the application of SPB not only serves as a source of soil DOC but also promotes the dissolution of soil organic matter, positively affecting soil DOC content. This may be related to microbial communities under appropriate exogenous addition concentrations. Due to the different properties of root surfaces and soils, microbes isolated from the rhizosphere, near‐rhizosphere, and far‐rhizosphere soils exhibit differences in species, physiology, and genetic microbial parameters.^[^ ^46^ ^]^ Rhizosphere effects drive microbial communities toward the near‐rhizosphere region, where plant root secretions and exudates provide a rich source of carbon for soil microbial communities.^[^ ^31b^ ^]^ Plant hormones in SPB play a role in shaping rhizosphere microbial communities and manipulating rhizosphere functions.^[^ ^47^ ^]^ IAA can promote the colonization of rhizosphere bacteria that enhance plant growth and serve as a source of carbon and nitrogen for rhizosphere bacteria.^[^ ^48^ ^]^ Similarly, SA and CK can also be utilized as carbon sources, promoting changes in soil community composition and microbial growth.^[^ ^49^ ^]^ Under certain environmental stress, soil microbes convert the precursor of auxin, L‐Trp, into nutrient release, thereby inducing microbial activity. Active microbial colonizers or r‐strategists are considered more likely to establish under these conditions.^[^ ^50^ ^]^

#### 2.3.2 Spatial Distribution of Nitrogen in Soil

Nitrogen application can regulate the carbon‐nitrogen metabolism and carbohydrate transport distribution in plants, thereby significantly impacting plant development.^[^ ^34^ ^]^ After applying fertilizer or co‐applying SPB, there was a significant increase in nitrogen content in various soil regions. In the rhizosphere, the total nitrogen content in the FC, LO, ME, and HI groups increased by 31.15%, 30.26%, 27.29%, and 6.4%, respectively, compared with the BC group (Figure 3a). This demonstrates the effectiveness of exogenous nutrient addition. There were no significant differences in total nitrogen content between the three extract addition groups and the FC group, with only the ME group exhibiting a 19.03% decrease in the rhizosphere region. However, their soil ammonium and nitrate nitrogen levels were significantly lower than those in the FC group, and as the extract concentration increased, the reduction in these levels also increased. In the far‐rhizosphere region, nitrate nitrogen decreased by 86.17%, 88.24%, and 92.29% in the three treatment groups, while ammonium nitrogen decreased by 11.64%, 31.69%, and 36.49%, respectively. This indicates that extract application can promote the conversion of soil nitrogen into organic nitrogen, favoring the accumulation of nitrogen elements in the soil. Particularly, elevated SPB concentrations typically facilitate the conversion of nitrogen into organic forms, promoting storage in the soil. The trend of chlorophyll changes further supports this view. Previous studies have found a close relationship between chlorophyll and nitrogen content in leaf tissues.^[^ ^51^ ^]^ There is a significantly positive relationship between chlorophyll concentration and nitrogen nutrition index, and soil nitrogen release and fertilization are key driving factors for chlorophyll concentration in leaves.^[^ ^52^ ^]^ As displayed in the leaf trait of Figure 3b, after applying active sludge extract, the chlorophyll content in the LO, ME, and HI groups increased significantly by 16.67%, 22.62%, and 28.57%, respectively, compared with the FC group. Therefore, SPB could contribute to improving nitrogen fertilizer agronomic efficiency and nitrogen absorption efficiency. The original active sludge contains numerous microbial groups related to nitrogen metabolism, hence, SPB contains a large number of rhizosphere microorganisms and their metabolites related to nitrogen element transformation. During SPB application, these microorganisms and their metabolites promote nitrogen transformation and utilization by transmitting chemical and biological information, resembling the effects of microbial agents.^[^ ^53^ ^]^

In the far rhizosphere region where plants could find it difficult to absorb and utilize nutrients, the soil could retain relatively primitive physicochemical properties, and nitrate nitrogen content was significantly lower than ammonium nitrogen. However, in the near rhizosphere region, the content of ammonium nitrogen was significantly lower than that of nitrate nitrogen. This indicated that although rice could absorb both forms of nitrogen, it could absorb ammonium nitrogen. In the FC, LO, ME, and HI groups, the ammonium nitrogen in the near rhizosphere was mainly absorbed and utilized, and its content was reduced by 84.86%, 93.36%, 96.96%, and 97.20% compared with the far rhizosphere. A specific purification enzyme on the surface of rice roots could convert ammonium nitrogen into electrically neutral ammonia and water in the presence of high concentrations of potassium ions in the soil. This process maintains a slightly acidic environment on the root surface, enhancing the absorption of ammonium nitrogen. In contrast to nitrate nitrogen, ammonium nitrogen has lower competitiveness with other nutrients, such as phosphorus, zinc, etc., allowing rice to absorb and utilize it more quickly and effectively. The co‐application of SPB can optimize the conversion and utilization of ammonium nitrogen by rice, and the optimization effect becomes more remarkable as the proportion of SPB increases.

### 2.4 Comprehensive Impact of Sludge‐Derived Plant Biostimulants on Rice

**Figure 4:**
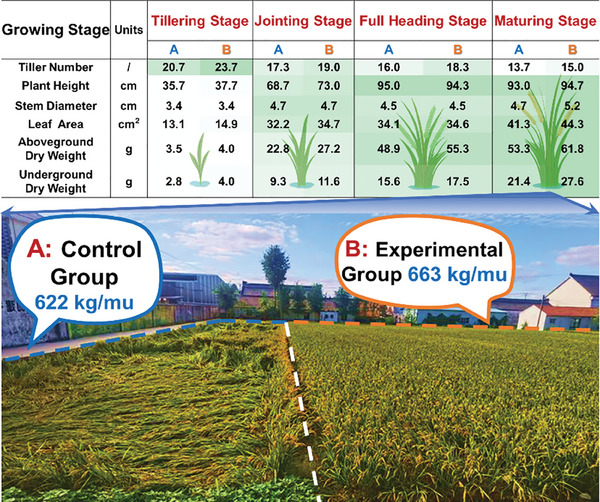
The regulation of sludge‐derived plant biostimulants application on the epigenetic performance of rice at different growth stages. Each experimental condition shown in the figure was performed with three biological replicates.

The optimization of soil physicochemical properties and the improvement of nitrogen utilization efficiency have led to superior development of rice in the SPB co‐application group compared with the BC and FC groups. The growth and development of rice, as well as its yield, are reflected by the accumulation of aboveground dry weight and belowground dry weight. The roots of rice in the underground part are the organs for absorbing water and nutrients, and their dry weight is closely associated with the construction and yield of aboveground organs. The impact of SPB on rice yield extends beyond reduced grain production, encompassing diminished quality and increased susceptibility to lodging and secondary infections. As illustrated in Figure 3b, the plant dry weight in the LO, ME, and HI groups significantly increased compared with the BC group, with aboveground dry weight increased by 15.49%, 33.80%, and 46.48%, and belowground dry weight increased by 25.53%, 34.04%, and 57.45%, respectively. Compared with the FC group with only fertilizer addition, the aboveground dry weight of LO, HE, and MI groups increased significantly by 10.81%, 28.38%, and 40.54%, and belowground dry weight increased significantly by 18%, 26%, and 48%. The aboveground part of rice is the main organ for photosynthesis and the production of photosynthetic products. An increase in leaf area is beneficial for enhancing leaf photosynthesis. Figure 3 demonstrates that compared with the BC group, the leaf area increased significantly in the LO, ME, and HI groups by 13.67%, 20.70%, and 21.12%, respectively. Compared with the FC group, these groups also exhibited significant increases of 11.91%, 18.84%, and 19.25% in leaf area. Plant hormone signals can transfer from soil and microorganisms to roots, travel through the xylem channels, and ultimately influence the structure, growth, and defense mechanisms of aboveground organs. These hormones typically interact or antagonize each other within the root‐soil continuum. For instance, SA and JA exhibited distinct responses to specific stresses; JA was negatively correlated with root biomass, while SA content exhibited a positive correlation with root biomass.^[^ ^43^, ^47^ ^]^ Overall, SPB has a beneficial impact on seedling growth, enhancing the accumulation of dry matter in the early growth stage of rice. The application of SPB could promote grain filling and increase the potential for rice yield increase. This was further confirmed by a one‐year field experiment (**Figure** **4**), where the rice yield in the experimental group with SPB spraying instead of fertilizer addition reached 663 kg mu^−1^, a 6.59% increase compared with the FC group (622 kg mu^−1^). Throughout the growth cycles of rice, measurements of tiller number, plant height, stem diameter, leaf area, aboveground dry weight, and underground dry weight exhibited superior shape indicators in the experimental group. The background of Figure 4 was taken post‐typhoon when the rice had reached the maturing stage. Notably, in the control group with fertilizer addition (left), significant lodging was observed in the rice plants, whereas the experimental group treated with SPB (right) demonstrated enhanced resistance to lodging stress. As SPB was applied to the same rice variety without reducing plant height, further comprehensive research is required to investigate SPB's effects on rice morphology, mechanical properties, gene expression, and other indicators related to lodging resistance under identical conditions.

### 2.5 Promotion Mechanisms of Sludge‐Derived Plant Biostimulants on Rice Growth

A “multi‐nutrient type biostimulant” was successfully developed for enhancing rice production using sewage sludge as a raw materials. 1) The composite of basic nutrient components was summarized: Sewage sludge, enriched with about half of the pollutants and resource materials from urban residents' sewage, contains essential nutrients, such as nitrogen, phosphorus, potassium, etc., providing fundamental nutritional support for plant growth. This innovative approach of converting waste into a valuable resource could provide a new paradigm for reducing chemical inputs in agricultural production. 2) The composite of effective plant stimulation components was summarized in the following: After ATH treatment, this study detected 51 plant hormones and 1177 metabolites for the first time, advancing the understanding of sewage sludge at a molecular level. This lays a crucial foundation for analyzing the ATH process metabolism of sludge and its high‐value utilization. 3) The composite of plant growth promotion benefits was summarized in the following: The findings indicated that appropriate concentrations of this biostimulant could promote rice seedling root growth, stabilize the activity of leaf antioxidant enzyme systems, and enhance the efficiency of nutrient ion absorption and nitrogen utilization by roots. These combined benefits provide initial research support for the widespread adoption of this biostimulant and further investigation into its mechanisms for enhancing efficiency.

**Figure 5:**
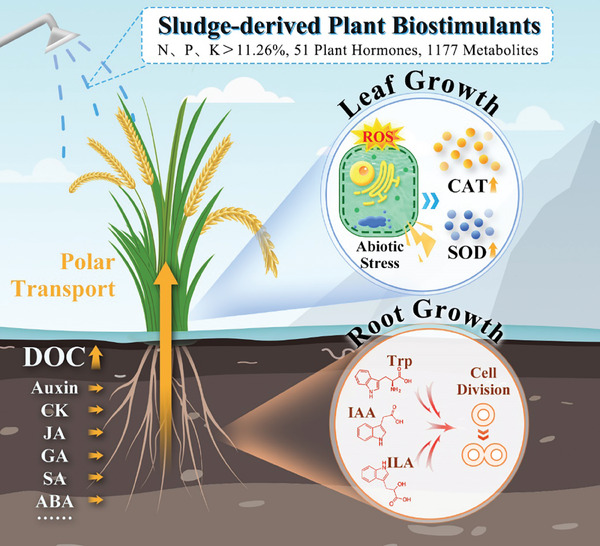
The stimulatory mechanism of sludge‐derived plant biostimulants on rice growth.

Specifically, the stimulatory mechanism of SPB on rice growth is illustrated in **Figure** **5**. Under appropriate concentration conditions of SPB application, the presence of growth hormones, such as IAA initiated rapid cell division in the initial cell group of rice roots, which was conducive to comprehensive improvement in indicators, such as root length, root surface area, root diameter, root volume, and root tip number. The growth and expansion performance of roots exhibited a significant advantage, and the polar transport of effective components could be enhanced. Moreover, the high content of two substances, L‐Trp and ILA, in SPB served as precursor materials for IAA synthesis, providing assurance for IAA slow release and sustained action. This led to an increase in the activities of catalase, superoxide dismutase, and reduced glutathione in rice leaves, effectively enhancing rice's defense against stress from reactive oxygen species and other free radicals on membrane lipids, maintaining internal balance in plants, and keeping cells at a normal level of metabolic activity. After application, SPB not only provided a source of DOC for the soil, but also, due to components, such as ABA, JA, SA, and CK, promoted the conversion of phosphorus and other affecting components in the soil to a state conducive to plant absorption, thereby enhancing rice's efficiency in utilizing nutrient ions and nitrogen. Additionally, post‐application, functional microbial recruitment in the rice rhizosphere soil could be induced, although this conclusion was hypothetically proposed in this study and requires further research confirmation supported by literature.

SPB influence plant growth and stress responses through several physiological and biochemical mechanisms. SPB provide essential nutrients such as nitrogen, phosphorus, and potassium, directly enhancing nutrient availability and uptake, while promoting root growth and development. Additionally, SPB contain natural plant hormones, such as auxins, cytokinins, and gibberellins that stimulate cell elongation, division, and nutrient mobilization, contributing to overall plant vigor. SPB also enhance stress tolerance through its rich antioxidant content, which neutralizes reactive oxygen species, and by upregulating stress‐related genes that produce protective molecules. Furthermore, SPB improve soil health by increasing organic matter and microbial activity, fostering beneficial microbial symbiosis that enhances nutrient uptake and protects against pathogens. It modulates metabolic pathways, improving photosynthesis and respiration efficiency, leading to higher biomass production and energy availability for growth and stress responses. Additionally, SPB induce the production of secondary metabolites such as flavonoids and phenolics, which bolster plant defense mechanisms. Finally, SPB aid in detoxifying harmful compounds by immobilizing heavy metals and breaking down organic pollutants, thus reducing their toxicity and adverse effects on plant health. These combined mechanisms contribute to healthier, more resilient, and productive crops.

The findings of the present study have significant practical implications for agricultural practices and policy‐making, which can contribute to more sustainable and efficient farming methods. First, the identification of key metabolites, such as proline and certain flavonoids that confer stress resistance can inform crop breeding programs aimed at developing varieties more resilient to environmental stresses, including drought, salinity, and pathogen attacks. For instance, metabolites involved in nitrogen assimilation, such as glutamine and glutamate, can be targeted to create crops that utilize nitrogen more efficiently, thereby reducing the need for chemical fertilizers. Additionally, metabolites, such as nicotine and specific phenolics, which play critical roles in plant defense mechanisms, can be enhanced to reduce reliance on chemical pesticides. This would improve overall crop health and reduce losses due to pests and diseases. Moreover, understanding the metabolic profiles related to fruit ripening and quality can inform optimal harvest times and post‐harvest storage methods. This will maximize nutrient content, flavor, and market quality while reducing post‐harvest losses. From a policy‐making perspective, our research supports the need for increased funding for research into plant metabolism. Policies that fund such research can accelerate advancements in agricultural technologies. Collaborative public‐private initiatives can facilitate the application of these findings to practical farming solutions, enhancing crop management and breeding strategies. Furthermore, insights from our study can inform policies that promote sustainable agricultural practices. For instance, incentivizing the use of crops with natural pest and disease resistance can reduce dependence on chemical inputs, promoting environmental sustainability. Understanding metabolites involved in nutrient uptake and stress responses can also lead to improved soil health and water management practices, which are crucial for long‐term agricultural sustainability. Lastly, recognizing the roles of metabolites in crop quality and nutritional value can guide the development of nutritional guidelines and quality assurance programs. This can ensure the cultivation and distribution of nutritionally superior crops, addressing public health concerns related to diet and nutrition while ensuring consistent quality and safety of agricultural produce. By integrating these findings into agricultural policies, we can promote a more sustainable, efficient, and health‐oriented agricultural system.

While this study provided valuable insights into the effects of SPB on plant growth and stress responses, there were several potential limitations to consider. First, the specific molecular mechanisms by which SPB influences plant physiology remained incompletely understood, necessitating further investigation. Additionally, the study's scope was limited to certain concentrations of SPB and specific growth stages of rice plants, which might not fully capture the broader applicability and long‐term effects of SPB across different plant species and environmental conditions. Future research should concentrate on comprehensive multi‐omics approaches, integrating genomics, proteomics, and metabolomics, to elucidate the detailed molecular pathways involved in SPB‐mediated plant growth and stress resilience. Longitudinal studies examining the effects of SPB over multiple growth cycles and in various soil types and climatic conditions will provide a more robust understanding of its efficacy and potential environmental impacts. Investigating the interaction between SPB and soil microbiomes, as well as the potential for SPB to mitigate soil and plant contamination by heavy metals and organic pollutants, would also be valuable. Lastly, field trials comparing SPB with conventional fertilizers under real agricultural conditions would help to validate laboratory findings and assess the practical benefits of SPB in sustainable agriculture.

## 3 Conclusion

In conclusion, this study represented a significant advancement in our understanding and application of SPB in crop production. By employing a combination of non‐targeted metabolomic qualitative and targeted metabolomic quantitative approaches, we have uncovered a comprehensive profile of 51 plant hormones and 1177 metabolites in SPB, unveiling the molecular intricacies of sludge ATH. The findings demonstrated that low concentrations of SPB exerted multiple beneficial effects on rice roots, leaves, and the root‐soil system. Specifically, the presence of hormones, such as IAA in rice roots could enhance root growth and expansion, leading to improvements in root morphology and function. Moreover, the enhanced activities of key antioxidant enzymes in rice leaves contributed to the improved stress tolerance, thereby bolstering plant resilience to environmental challenges. Furthermore, SPB could not only enriche the soil with dissolved organic carbon, but also facilitate the transformation of essential nutrients, such as phosphorus, into plant‐available forms, enhancing nutrient uptake efficiency by rice. These findings highlighted the potential of SPB to enhance crop productivity while promoting soil health and sustainability in agricultural systems. By providing a robust theoretical framework for understanding the metabolic dynamics of sludge ATH and the efficacy of SPB in land use optimization, this study may lay the basis for the widespread adoption of SPB as a promising tool for sustainable agriculture.

## 4 Experimental Section

### Preparation of Sludge‐Derived Plant Biostimulants

The SPB were produced from dewatered sludge collected from a municipal sewage treatment plant in Jiangsu (China), containing 20.12% (w/w) total solid (TS) content and 32.69% (w/w) volatile solid (VS) of TS. CaO was added in a TS ratio of 1:5 (w/w) to dewatered sludge to create an alkaline environment (pH = 12.5 ± 0.5). After 4 h reaction in a closed pressure‐resistant hydrolysis device (effective volume 200 L) at 120 °C,^[^ ^14^ ^]^ the dehydrated liquid was collected as the precursor of SPB by means of plate and frame pressure filtration. Further filtration (0.45 µm) removed suspended particles to obtain clarified SPB, and physiochemical and security properties of raw sludge and SPB are shown in Table 2.

### Experimental Design for Seed Germination and Hydroponics

Rice seeds (japonica rice Nangeng 9108) were selected based on their relatively uniform size and plumpness. The surface of the seeds was disinfected by soaking them in a 2% w/v solution of sodium hypochlorite for 30 min. The seeds were thoroughly rinsed with distilled water until the pungent smell disappeared. The prepared seeds were then divided into two groups for germination and hydroponics experiments, respectively.

Seeds designated for the germination experiment were placed in a light‐avoiding germination chamber at a constant temperature of 25 °C. Seeds with exposed embryos and consistent growth were placed in Petri dishes containing two layers of qualitative filter paper (6 cm in diameter), involving 20 seeds per dish. A single‐factor controlled variable experiment was conducted using SPB concentration, comprising one blank group (containing only distilled water) and five experimental groups (0.33%, 1%, 3.3%, 10%, 100%). Each dish received 10 mL of the respective extract or distilled water. Three replicates were set up for each group. The petri dishes were covered and incubated in the light‐avoiding germination chamber at 25 ± 1 °C for 5 days. The germination status of the seeds was recorded daily, and the root length was measured. The germination index (GI) of the seeds was calculated as follows (Equation 1):

(1) $$\mathrm{GI}=\sum \frac{G_{t}}{D_{t}}\;$$

where G*_t_* represents the daily number of germinated seeds during the germination experiment, D*_t_* represents the number of days of germination and ∑ represents the sum.

The seeds used for the hydroponics experiments were soaked in pure water and placed in a refrigerator at 4 °C for 24 h. Seedling trays were covered with gauze and filled with deionized water, and the seeds were evenly distributed on the gauze. Once germination occurred and the primary roots of the rice seedlings reached a length of 4–6 cm (≈6 days), seedlings with consistent growth were selected. A sponge was gently wrapped around the lower hypocotyl of the seedlings, and their roots were passed through circular holes in a hydroponic tray filled with nutrient solution. The hydroponic tray, made of polyethylene, measured 32 × 17.5 × 5 cm^3^ and had a black outer surface to prevent light exposure. The nutrient solution consisted of Hoagland's macronutrient solution and Arnon's micronutrient solution. Using SPB concentration as a single‐factor controlled variable, one blank group (containing only distilled water) and four experimental groups (0.04%, 0.06%, 0.11%, and 0.17%) were set up. Each group had four replicates, with six rice seedlings in each replicate. The light intensity was set at 300–320 µmol·(m^2^·s)^−1^, relative humidity of 65–80%, and a photoperiod of 16 h of light followed by 8 h of darkness. The temperature was maintained at 22 °C. The seedlings were cultured continuously for 30 days, with the nutrient solution changed every 2 days. On the 10th, 20th, and 30th days, samples were collected from the aboveground parts and roots for measurements of dry weight, fresh weight, root morphology, root vitality, and enzyme activity.

### Experimental Design for Root Box

Root box experiments were conducted to investigate the growth and development of rice roots. The soil was collected from the topsoil (0–20 cm) of paddy fields in Anhui experimental base, China. The soil was purified by removing branches, leaves, stones, and other impurities, air‐dried, ground, sieved through a 20‐mesh sieve, and packed into custom‐made root boxes (Figure S2, Supporting Information, dimensions: 23 cm long, 18 cm wide, 23 cm high) with nylon cloth separating the root compartment into near‐rhizosphere and far‐rhizosphere areas. Each root box contained 5.0 kg of soil (dry weight). The initial physical and chemical properties of the soil used were summarized as follows: pH 5.7, soluble organic carbon content 2.7 mg kg^−1^, electrical conductivity 105 µs cm^−1^, nitrate nitrogen 3.58 mg kg^−1^, and ammonium nitrogen 14.52 mg kg^−1^.

Five treatment groups were established, including blank control (BC, no fertilizer application), fertilizer control (FC, fertilizer application only), low SPB blending (LO, 30% nitrogen reduction by fertilizer + 2 mL of SPB), medium SPB blending (ME, 30% nitrogen reduction by fertilizer + 4 mL of SPB), and high SPB blending (HI, 40 mL of SPB as the sole nitrogen source + phosphorus and potassium). Urea, potassium dihydrogen phosphate, and potassium chloride were used as nitrogen, phosphorus, and potassium sources in the fertilizers, respectively. Three replicates were set up for each treatment group.

The greenhouse was naturally lit and maintained at 25 ± 1 °C with humidity ranging from 8% to 14%. After rice seedlings had grown to three leaves and one heart stage, uniformly growing seedlings were selected and transplanted into root boxes, with four plants per root box. From transplanting until sampling, each root box maintained a shallow water layer of 1–2 cm. Soil samples were collected from rice seedlings, the root compartment, near‐rhizosphere, and far‐rhizosphere after 30 days of continuous incubation for subsequent determination of growth and leaf traits, as well as soil physicochemical parameters (pH, DOC, conductivity.

### Methods for Detection of Plant Hormones

Take 50 µL of the SPB sample, add 10 µL of a 100 ng mL^−1^ internal standard mixed solution, mix well, evaporate to dryness, reconstitute the dried residue with 100 µL of 80% methanol/water solution, filter through a 0.22 µm membrane, transfer to a sample vial, and use for UPLC‐MS/MS analysis.^[^ ^54^ ^]^

The UPLC system (ExionLC AD) uses a Waters ACQUITY UPLC HSS T3 C18 column (1.8 µm, 100 mm × 2.1 mm i.d.) as the chromatographic column. The mobile phase consists of solvent A, which is ultrapure water with 0.04% acetic acid, and solvent B, which is acetonitrile with 0.04% acetic acid. The flow rate is set at 0.35 mL min^−1^, with a column temperature of 40 °C, and an injection volume of 2 µL. The gradient elution program is as follows: 0 min, A/B is 95:5 (V/V); 1.0 min, A/B is 95:5 (V/V); 8.0 min, A/B is 5:95 (V/V); 9.0 min, A/B is 5:95 (V/V); 9.1 min, A/B is 95:5 (V/V); 12.0 min, A/B is 95:5 (V/V).^[^ ^55^ ^]^

The electrospray ionization (ESI) source temperature of the tandem mass spectrometer (QTRAP 6500 +) was set at 550 °C. In positive ion mode, the mass spectrometer voltage was 5500 V, while in negative ion mode, the mass spectrometer voltage was –4500 V. The curtain gas (CUR) pressure was maintained at 35 psi. In the QTRAP 6500 +, each ion pair was scanned and detected based on optimized parameters including declustering potential (DP) and collision energy (CE).^[^ ^56^ ^]^

The Metware Database (MWDB) was constructed based on standard compounds along with the standard curve (external ± isotopic internal standard) exhibiting a linear relationship of 0.99 or higher. Mass spectrometry data was analyzed using Analyst 1.6.3 software for preprocessing to generate total ion chromatograms and extracted ion chromatograms. Using the retention time and peak information from reference standards, MultiQuant 3.0.3 software was employed to integrate and calibrate chromatographic peaks detected in different samples, ensuring accuracy in qualitative and quantitative analysis.

The integrated peak area ratios of all detected samples were substituted into the linear equation of the standard curve (Equation 2) for calculation. Subsequently, the calculated values were further processed using the calculation formula to obtain the actual content data of the substance in the samples.

(2) $$\text{Hormone Content}\;\left(\mathrm{ng}/\mathrm{mL}\right)=c*V_{1}/1000/V_{2}$$

where c represents the concentration value obtained by substituting the integrated peak area ratio into the standard curve (ng mL^−1^), V_1_ represents volume of the solution used for reconstitution (µL), and V_2_ represents volume of the sample taken (mL).

### Methods for Detection of Widely Targeted and Non‐targeted Metabolites

In the present study, the newly prepared SPB samples were immediately frozen in liquid nitrogen and stored at −80 °C. They were then placed in a freeze dryer (Scientz‐100F) for vacuum freeze‐drying. Using a grinder (MM 400, Retsch) at 30 Hz for 1.5 min, the samples were ground into a fine powder. 50 mg of the sample powder was weighed and mixed with 1200 µL of 70% methanol‐water internal standard extraction solution pre‐cooled to −20 °C. The mixture was vortexed every 30 min for 30 s each time, for a total of six vortexing cycles. After centrifugation at 12 000 rpm for 3 min, the supernatant was collected, filtered through a microporous membrane (0.22 µm pore size), and stored in sample vials for LC‐MS/MS analysis. Ultra‐Performance Liquid Chromatography (UPLC) (ExionLC AD) coupled with tandem mass spectrometry (MS/MS), equipped with an Agilent SB‐C18 (1.8 µm, 2.1 mm × 100 mm) chromatographic column, was used for data acquisition. Each injection volume was 2 µL, with a column temperature of 40 °C. The mobile phases consisted of ultrapure water with 0.1% formic acid (Phase A) and acetonitrile with 0.1% formic acid (Phase B). The gradient elution was set at a flow rate of 0.35 mL min⁻¹, starting with 5% B phase, linearly increasing to 95% B phase over 9 min, maintained at 95% for 1 min, then returning to 5% B phase and held constant for 14 min. The mass spectrometer's electrospray ionization source temperature was set at 550 °C, with ion spray operated at a positive ion voltage of 5500 V or a negative ion voltage of −4500 V. The ion source gas I (GSI) was set at 50 psi, gas II (GSII) at 60 psi, and CUR at 25 psi. QQQ scanning used MRM mode, with collision gas (nitrogen) set at moderate levels. Further optimization of DP and CE was done for each MRM ion pair. Substance identification was based on secondary spectrum information in the self‐built MWDB, removing isotope signals, repeated signals of K⁺ ions, Na⁺ ions, NH₄⁺ ions, and fragment ions of larger molecular weight substances.

For targeted metabolomic analysis, 50 µL of the SPB sample was added to 10 µL of a 100 ng mL^−1^ internal standard mixed solution, mixed well, evaporated to dryness and reconstituted with 100 µL of 80% methanol/water solution. The mixture was filtered through a 0.22 µm membrane and transferred to a sample vial for UPLC‐MS/MS analysis. The UPLC system (ExionLC AD) used a Waters ACQUITY UPLC HSS T3 C18 column (1.8 µm, 100 mm × 2.1 mm i.d.). The mobile phase consisted of ultrapure water with 0.04% acetic acid (solvent A) and acetonitrile with 0.04% acetic acid (solvent B). The flow rate was set at 0.35 mL min^−1^, with a column temperature of 40 °C, and an injection volume of 2 µL. The gradient elution program started with A/B at 95:5 (v/v), changed to 5:95 (v/v) over 8 min, maintained at 5:95 (v/v) for 1 min, then returned to 95:5 (v/v) and held for 3 min. The electrospray ionization (ESI) source temperature of the tandem mass spectrometer (QTRAP 6500 +) was set at 550 °C, with a positive ion mode voltage of 5500 V and a negative ion mode voltage of −4500 V. The curtain gas (CUR) pressure was maintained at 35 psi. Each ion pair was scanned and detected based on optimized parameters including declustering potential (DP) and collision energy (CE). The Metware Database (MWDB) was constructed based on standard compounds, with the standard curve exhibiting a linear relationship of 0.99 or higher. Mass spectrometry data were analyzed using Analyst 1.6.3 software for preprocessing to generate total ion chromatograms and extracted ion chromatograms. Using retention time and peak information from reference standards, MultiQuant 3.0.3 software was employed to integrate and calibrate chromatographic peaks detected in different samples, ensuring accuracy in qualitative and quantitative analysis. The integrated peak area ratios were substituted into the linear equation of the standard curve (Equation 2) for calculation and further processed to obtain the actual content data of the substances in the samples.

### Earthworm Acute Toxicity Test

Healthy red earthworms of 2 months of age or older with ring bands and 300–600 mg body weight were selected for the test. Before the start of the toxicity test, the earthworms were checked for susceptibility with chloroacetamide according to the method of OECD207,^[^ ^57^ ^]^ and earthworms with 14 d‐LC50 values of chloroacetamide in the range of 10–50 mg kgdw^−1^ were selected for the toxicity test.

In the seed germination experiment, SPB concentrations used were 0.33%, 1%, 3.3%, 10%, and 100%, with a blank control group containing only distilled water. Each petri dish, containing 20 seeds, was supplemented with 10 mL of the respective extract or distilled water, and three replicates were set up for each group. The germination status of the seeds was observed daily, and the root length and germination index (GI) were recorded after 5 days of incubation.

In the hydroponics experiment, the SPB concentrations used were 0.04%, 0.06%, 0.11%, and 0.17%, with a blank control group containing only distilled water. Each group had four replicates, with six rice seedlings in each replicate. The seedlings were cultured for 30 days, with the nutrient solution changed every 2 days. Measurements of dry weight, fresh weight, root morphology, root vitality, and enzyme activity were taken on the 10th, 20th, and 30th days of the experiment. In the root box experiment, five treatment groups were set up: Blank control (BC, no fertilizer application), Fertilizer control (FC, fertilizer application only), Low SPB blending (LO, 30% N reduction by fertilizer + 2 mL of SPB), Medium SPB blending (ME, 30% N reduction by fertilizer + 4 mL of SPB), and High SPB blending (HI, 40 mL of SPB only as N source + phosphorus and potassium). Soil samples were collected from the root compartment, near‐rhizosphere, and far‐rhizosphere after 30 days of continuous incubation to determine growth and leaf traits, as well as soil physicochemical parameters (pH, DOC, conductivity).

Seven concentration gradients were set: 0, 20.6, 61.7, 185, 556, 1667, and 5000 mg kg^−1^ (according to the results of the pre‐test, the maximum concentration of 5000 mg kg^−1^ was chosen as the highest concentration because the mortality rate was less than 50%). Each group was tested in a 1 L beaker with 750 g (wet weight) of artificial soil and 10 earthworms in four parallel settings. The beakers were covered with film and holes were punched in the film and placed in a climatic chamber with continuous light (14 d period, 20 °C, 75% humidity). The average weight and number of surviving earthworms were recorded at 0 and 14 d. The response of the anterior tail of the earthworms to mechanical stimulation was examined at 7 and 14 d, respectively.

### Statistical Analysis

Data obtained from the experiments were subjected to comprehensive statistical analysis to ensure a robust interpretation of the results. Prior to analysis, all data underwent pre‐processing steps, including normalization and transformation where applicable. Outliers were identified and evaluated using appropriate statistical methods. Data are presented as mean ± standard deviation (SD), with sample sizes (n) indicated for each statistical analysis. Statistical significance was determined using a variety of methods depending on the nature of the data and experimental design. For comparisons between groups, one‐way analysis of variance (ANOVA) followed by Tukey's post‐hoc test was employed. Two‐sided testing was conducted, and significance was set at the 5% level (alpha = 0.05). Assumptions of normality and homogeneity of variances were assessed using appropriate tests such as Shapiro‐Wilk and Levene's tests, respectively. In cases where multiple comparisons were made, adjustments were applied to control the family‐wise error rate, such as the Bonferroni correction. Additionally, the validity of assumptions underlying the chosen statistical tests was confirmed. Statistical analysis was performed using SPSS 27.0 software (IBM, Armonk, NY, USA).

## Conflict of Interest

The authors declare no conflict of interest.

## Supporting information



Supporting Information

## Data Availability

The data that support the findings of this study are available from the corresponding author upon reasonable request.
