# Supplementary material for: Elucidating the Molecular Mechanisms and Comprehensive Effects of Sludge‐Derived Plant Biostimulants on Crop Growth: Insights from Metabolomic Analysis
Source: Adv Sci (Weinh). 2024 Nov 14;12(2):2404210. doi: 10.1002/advs.202404210 (PMC11727372; doi:10.1002/advs.202404210)
Supplement: Supplementary file 1 — Supporting Information [file ADVS-12-2404210-s001.docx]

Supporting Information

**Elucidating the Molecular Mechanisms and Comprehensive Effects of Sludge-Derived Plant Biostimulants on Crop Growth: Insights from Metabolomic Analysis**

Yu Hua ^#^, Shuxian Chen ^#^, Tong Tong, Xiaoou Li, Rongting Ji *, Qiujin Xu *, Yue Zhang, Xiaohu Dai *

(^#^ Equally contributing author)

**Figure S1.** A Total of 1,177 Metabolites and Their Classification Detected from Sludge-derived Plant Biostimulants.

**Figure S2.** The Custom-made Root Box Used in This Study.
